# Mortality in women with a history of incarceration in Norway: a 20-year national cohort study

**DOI:** 10.1093/ije/dyae032

**Published:** 2024-03-13

**Authors:** Vegard G Svendsen, Anne Bukten, Torbjørn Skardhamar, Marianne Riksheim Stavseth

**Affiliations:** Norwegian Centre for Addiction Research, SERAF, University of Oslo, Oslo, Norway; Norwegian Centre for Addiction Research, SERAF, University of Oslo, Oslo, Norway; Division of Mental Health and Addiction, Oslo University Hospital, Oslo, Norway; Department of Sociology and Human Geography, University of Oslo, Oslo, Norway; Norwegian Centre for Addiction Research, SERAF, University of Oslo, Oslo, Norway; Division of Mental Health and Addiction, Oslo University Hospital, Oslo, Norway

**Keywords:** Prison, mortality, women’s health, at-risk populations, substance use, non-communicable disease

## Abstract

**Background:**

Women carry a substantial burden of psychiatric, somatic and lifestyle-related morbidity in the prison context. By describing causes of death and estimating the risk and burden of mortality compared with the general population, this study investigates how mortality operates in this highly marginalized and under-researched population.

**Methods:**

In this registry-based study of all women incarcerated in Norwegian prisons from 2000 to 2019 (*N* = 11 313), we calculated crude mortality rates, years of lost life and, by using mortality in age-matched women from the general population as a reference, age-standardized mortality ratios and years of lost life rates.

**Results:**

Over a mean follow-up time of 10.7 years, at a median age of 50 years, 9% of the population had died (*n* = 1005). Most deaths (80%) were premature deaths from an avoidable cause. Drug-induced causes and deaths from major non-communicable diseases (NCDs) were most frequent (both 32%). Compared with women in the general population, women with a history of incarceration were more likely to die from any cause. Trends in annual age-standardized years of lost life rates suggest that the mortality burden associated with major NCDs has gradually replaced drug-induced causes.

**Conclusions:**

Women with a history of incarceration die at a greater rate than their peers and largely from avoidable causes. The profile of causes contributing to the substantial burden of mortality placed on this population has changed over time and has important implications for future efforts to reduce morbidity and the risk of premature death following release from prison.

Key Messages

- The majority (80%) of deaths among women with a history of incarceration in Norway were premature and from an avoidable cause.
- Compared with age-matched controls from the general population, women with a history of incarceration were five times more likely to die from any cause and nine times more likely to die prematurely from a preventable cause of death.
- Even though <0.05% of the Norwegian adult female population ever spends time in prison, we found that 13% of all deaths from HIV or hepatitis, 11% of all drug-induced deaths and 5% of all alcohol-related deaths that occurred in any women in Norway between 2000 and 2019 were among women who had also been to prison during the same period.
- As among women in the general population, the burden of drug-induced mortality has decreased among women with a history of incarceration.
- Although the mortality burden associated with alcohol-related deaths and major non-communicable diseases has decreased in the general population, it has increased among women with a history of incarceration and has replaced drug-induced deaths as the leading contributor to premature mortality.

## Introduction

Women carry a disproportionally large burden of morbidity in the prison context.^1–3^ They are more likely than men to have chronic somatic disorders,^4^^,^^5^ HIV or hepatitis C,^6^^,^^7^ mental health problems and substance use disorders;^8^^,^^9^ and rates of comorbid conditions and other complex health problems are high.^10–12^

Compared with women in the general population, women with a history of incarceration are more likely to smoke, drink and use drugs excessively;^13^^,^^14^ to be overweight or obese;^15^ to be overdue for cervical cancer screening;^16^ to have poor oral health;^17^ and to present with life histories involving trauma and victimization.^18^

Furthermore, women are a minority within prisons and subject to gender-specific stigmas surrounding drug use, criminal behaviour and incarceration. Thus, they are less likely to receive gender-informed care and more likely to encounter social barriers to successful rehabilitation and reintegration.^19–22^

The already high rates of substance use and non-communicable diseases (NCDs) among people in prison^23^^,^^24^ combined with the added risk of poor health and poor health outcomes among incarcerated women represent a public health concern and warrant a closer monitoring of mortality in this population.^25^

However, there is a shortage of recent studies reporting on all-cause mortality among women with a history of incarceration, especially from the European context. Among the relatively few studies investigating mortality risk relative to the general population [such as standardized mortality ratios (SMRs)],^26–28^ many do not report gender-specific results.^29–33^ This is of some concern as there are likely important gender differences in mortality^26^^,^^30^^,^^34^^,^^35^ and in the association between incarceration and premature death.^20^

Greater attention to gender differences in the prison context is essential.^36^ A gendered perspective could potentially guide the implementation of targeted public health interventions and treatment strategies^37^ to more effectively prevent premature deaths and improve health-related quality of life among women with a history of incarceration.^38^^,^^39^

To the best of our knowledge, this is the first original study in the epidemiological prison literature to focus exclusively on mortality among women. The primary aim of this study is to investigate the causes and burden of death among women with a history of incarceration and to estimate risk of death in this population compared with women in the general population. We also want to explore potential temporal trends in the burden associated with leading causes of death.

## Methods

### Study population and data sources

This study from Norway (see Supplementary Information, available as Supplementary data at *IJE* online) is based on the PriSUD-project^40^ using data from the Norwegian Prison Release Study (nPRIS),^41^ which is based on data from the Norwegian prison registry^42^^,^^43^ linked to various national registries. All women holding a personal identification number (PIN), serving a prison sentence or pre-trial detention between 1 January 2000 and 31 December 2019 (*n* = 11 313) were included. Sentences served in the community (e.g. home detention or community service) were not included in the data set.

The nPRIS cohort was linked to the Norwegian cause-of-death registry (NDR)^44^ using the PIN. Together, this data material provided individual-level information about demographics and sentencing (e.g. age, sentence length, number of sentences) and mortality (e.g. date and cause of death).

From the NDR, we also had access to population-level mortality (aggregated by 5-year age groups), including cause of death, from the adult (≥15 years) female population. These data allowed comparison of mortality in women with a history of incarceration with that in women in the general population.

### Mortality measures

Causes of death were coded according to the International Statistical Classification of Diseases and Related Health Problems—10th Revision (ICD-10).^45^ Causes of death were categorized as all-cause mortality (including from unknown causes), avoidable premature mortality before age 75 years,^46^ mortality from substance use (drug-induced and alcohol-related deaths^47^) and major NCDs (i.e. cardiovascular disease, cancer, diabetes mellitus and chronic respiratory disease)^48^ and by ICD-10 chapters (Table 1). As is particularly relevant for this group, we also report mortality from causes strongly associated with smoking (i.e. lung and laryngeal cancer and chronic respiratory disease), as well as death from cervical cancer and HIV and hepatitis (see Supplementary Information, available as Supplementary data at *IJE* online).

**Table 1.** Definitions of included cause-of-death categories based on ICD-10 codes

Category	Definition
All-cause mortality	Any recorded death
Avoidable premature mortality	
Deaths from preventable causes^a^	(A00–09, 15–19, 33–37, 39, 40.3, 41.3, 49.2, 50–60, 63–64, 80), (B01, 05, 06, 15–24, 50–54, 90), (C00–09, 10–16, 22. 33–34, 43, 45, 67, 53), (D50–53), (E10–14, E244), (F10–19), (G000–001, 312, 621, 721), (I10–13, 15, 20–25, 42.6, 60–71, 73.9), (J09–14, 40–44, 60–70, 82, 92), (K29.2, 70, 73, 74.0–74.2, 74.6, 85.2, 86.0), (Q00–01, 05, 860), (R78.0), (U07.1–07.2), (V00–W30), (X40–99), (Y00–34)
Deaths from treatable causes^a^	(A40.0–40–2, 40.8–41.2, 41.4–41.5, 41.8–41.9, 38, 46, 48.1, 49.1, 50–60, 64–64), (B01, 05–06, 15–24, 50–54), (C18–21, 50, 54–55, 62, 73, 81, 91.0–91.1), (D10–30), (E0, 24.0–24.3, 24.8–25, 27), (G00.0–0.03, 0.08–0.09, 03, 40–41), (I0, 26, 80, 82.9), (J0, 12, 15–18, 20–22, 45–47, 80–81, 85–86, 90, 93–94), (K25–28, 35–38, 40, 80–83, 85.0–85.3, 85.8–85.9, 86.1–86.3, 86.8–86.9), (L03), (N13, 17–19, 20–21, 23, 25–27, 34.1, 35, 40, 70–73, 75.0–75.1, 76.4, 76.6), (O00–99), (P00–99), (Q20.0–20.6, 20.8), (Y40–70, 80–84)
Mortality from substance use and non-communicable disease	
Drug-induced deaths	(F11–16, 19, 55), (X40–44, 60–64, 85), (Y10–14)
Alcohol-related deaths	(F10), (X45), (Y15), (E24.4), (G31.2, 62.1, 72.1), (R78.0), (I426), (K29.2, 70.0–70.4, 70.9, 85.2, 86.0)
Major non-communicable diseases^b^	(C00–97), (E10–14), (I00–99), (J40–47)
Smoking-related deaths	(C32–34), (J40–47)
Cause-specific mortality (by ICD-10 chapters)	
I. Certain infectious and parasitic diseases	(A00–B99)
HIV or hepatitis c	(B15–24)
II. Neoplasms	(C00–D48)
Cancer^b^	(C00–97)
Lung or laryngeal cancer^c^	(C32–34)
Cervical cancer	(C53)
V. Mental and behavioural disorders	(F00–99)
Due to drug use	(F11–16, 18–19)
Due to alcohol use	(F10)
IX. Diseases of the circulatory system^b^	(I00–99)
X. Diseases of the respiratory system	(J00–99)
Chronic respiratory disease^b^^c^	(J40–44)
XI. Diseases of the digestive system	(K00–93)
Alcohol-related	(K29.2, 70.0–70.4, 70.9, 85.2, 86.0)
Diabetes mellitus^b^	(E10–14)
XVIII. Symptoms, signs and abnormal clinical and laboratory findings, not elsewhere classified	(R00–99)
III–IV/VI–VIII. Other non-external causes and unknown causes	(D50–89), (E00–90), (G00–G99), (H00–95), Unknown cause
XX. External causes of morbidity and mortality	(V01–Y98)
Accidents and poisonings with unknown intent	(V01–X59)
Drug poisoning	(X40–44), (Y10–14)
Alcohol poisoning	(X45), (Y15)
Homicide	(X85–Y09)
Suicide	(X60–84)
Intentional drug poisoning	(X60–64)

ICD-10, International Statistical Classification of Diseases and Related Health Problems (10th Revision).

a In persons aged <75 years.

b Global leading cause of non-communicable disease (as defined by the World Health Organization).

c Smoking-related.

Given the relatively small sample size and because alcohol-related mortality is typically not included when reporting deaths from substance use in the prison context, when grouping leading causes of death, we combined alcohol-related mortality with major NCDs. Results from a sensitivity analysis combining alcohol-related mortality with drug-induced causes is presented in Supplementary Figure S1 (available as Supplementary data at *IJE* online).

To avoid double counting, deaths recorded in our study cohort were subtracted from deaths in the general population data.

### Statistical methods

Crude mortality rates (CMRs) were calculated as the number of deaths divided by the days at risk (in person years) multiplied by 100 000, reported with 95% CIs. Time at risk for each individual was defined as days since the date of the first recorded entry into prison until the date of death or end of observation (31 December 2019).

To identify the risk of death among women with a history of incarceration in comparison with age-matched women in the general adult population, we calculated SMRs. The SMRs were derived by comparing the total number of observed deaths in the study cohort with the expected number of deaths, assuming the study cohort had the same age-specific death rate as the general adult female population (grouped by 5-year age intervals).

The burden of premature death was assessed using the years of lost life (YLL) methodology, as described by Martinez *et al*,^49^ and standardized life expectancy (SLE) defined by the 2015 Global Burden of Disease study.^50^ YLL is the sum of the lost life years and is calculated by multiplying the number of observed deaths by the corresponding SLE within each (5-year) age category. We calculate the annual age-standardized years of life lost rate (ASYR) by calculating the YLL as a rate per 100 000 observations (i.e. number of persons at risk times 100 000) in each age category by year and weighting this rate by a standard population, in this case the age distribution of women in the Norwegian general population as reported for each year in census data from Statistics Norway.^51^

To explore temporal trends in the burden of premature death, we calculated the annual ASYR for all-cause mortality in both the prison and the general population, as well as separately for (i) drug-induced causes and (ii) alcohol and major NCDs. Using local polynomial regression, a non-parametric expansion on the standard least-squares approach in which points are fitted locally based on the proximity (weighted by distance) to surrounding data points, locally estimated scatterplot smoothing lines (LOESS) were added to the annual ASYRs.^52^

As the ASYR is a product of both the number of observed deaths and the age of those who died, at least three underlying mechanisms could be driving any observed changes over time: (i) women could be dying less or more frequently from said cause; (ii) women could be dying later or earlier in life from said cause; or (iii) some combination of (i) and (ii).

## Results

Between 2000 and 2019, 11 313 women served time in a Norwegian prison, entering prison at a median age of 34 years (Table 2). The median sentence length was ∼1 month (Table 2 and Supplementary Table S1, available as Supplementary data at *IJE* online). During the observation period, and over a mean follow-up time of 10.7 years, ∼9% (*n* = 1005) of the population had died at a median age of 50 years (Table 3). The majority of deaths occurred outside of prison. Fourteen deaths occurred during incarceration (of which six were in prison and eight occurred while on suspension or at a treatment facility outside of prison).

**Table 2.** Cohort characteristics

Characteristic	Value
Number of women incarcerated between 2000 and 2019	11 313
Age in years at first recorded entry to prison, median (IQR)	34 (26–43)
Sentence length in days, median (IQR)	27 (18–59)
Women with history of two or more incarcerations in the study period	2656 (23%)
Observation time in person-years, mean (SD)	10.7 (5.7)
Women who died during observation period	1005 (8.9%)

IQR, interquartile range.

**Table 3.** Mortality statistics for all-cause mortality, avoidable premature mortality and mortality from substance use and non-communicable diseases

Category	Number of prison population (%)	Median age (years)	Number of general population (%)	CMR (95% CI)	SMR (95% CI)	YLL (%)
All-cause mortality	1005 (100)	50	426 267 (100)	827.19 (776.05, 878.33)	5.17 (5.01, 5.34)	42 646.48 (100)
Avoidable premature mortality						
Deaths from preventable causes^a^	737 (73.33)	48	11 154 (26.17)	606.61 (562.81, 650.4)	8.98 (8.65, 9.32)	32 799.42 (76.91)
Deaths from treatable causes^a^	62 (6.17)	53	38 914 (9.13)	51.03 (38.33, 63.73)	2.01 (1.75, 2.27)	2397.53 (5.62)
Mortality from substance use and NCDs						
Drug-induced deaths (excluding alcohol)	321 (31.94)	41	2527 (0.59)	264.21 (235.3, 293.11)	36.39 (34.36, 38.42)	16 339.56 (38.31)
Alcohol-related deaths	89 (8.86)	54	1718 (0.40)	73.25 (58.03, 88.47)	24.48 (21.89, 27.08)	3408.66 (7.99)
Major NCDs^b^	320 (31.84)	58	272 223 (63.86)	263.38 (234.53, 292.24)	2.44 (2.30, 2.58)	11 035.07 (25.88)
Smoking-related	106 (10.55)	61	37 198 (8.98)	87.24 (70.63, 103.85)	3.82 (3.45, 4.19)	3436.94 (8.06)

CMR, crude mortality rate; NCD, non-communicable disease; SMR, standardized mortality ratio; YLL, years of life lost.

a In persons aged <75 years.

b Global leading cause of non-communicable disease (as defined by the World Health Organization).

The majority (*n* = 799; 80%) of observed deaths were premature deaths from an avoidable cause that was either preventable (73%) or treatable (6%) (Table 3). Drug-induced causes (*n* = 321, CMR: 264.21, 95% CI: 235.3–293.11) and deaths from major NCDs (*n* = 320, CMR: 263.38, 95% CI: 234.53–292.24) were equally frequent; each accounted for 32% of all observed deaths. Alcohol-related deaths (*n* = 89; 9%; CMR: 73.25, 95% CI: 58.03—88.47) also contributed substantially to overall mortality. One in three deaths from a major NCD was from a smoking-related cause (*n* = 106). One in 10 deaths was a suicide (*n* = 95; 9.5%) (Table 4).

**Table 4.** Mortality statistics for causes reported according to ICD-10 chapters, presented with relevant subcategories

Cause-specific mortality (by ICD-10 chapters)	Number of prison population (%)	Median age (years)	Number of general population (%)	CMR (95% CI)	SMR (95% CI)	YLL (%)
I. Certain infectious and parasitic diseases	28 (2.79)	51.5	9041 (2.12)	23.05 (14.51, 31.58)	12.23 (9.92, 14.54)	1081.39 (2.54)
HIV or hepatitis	20 (1.99)	51.5	129 (0.03)	16.46 (9.25, 23.68)	51.6 (40.07, 63.14)	779.73 (1.83)
II. Cancer^a^^c^	169 (16.82)	59	99 849 (23.42)	139.1 (118.13, 160.07)	1.77 (1.63, 1.9)	5737.40 (13.45)
Lung or laryngeal cancer^b^	64 (6.37)	62	17 531 (4.11)	52.68 (39.77, 65.58)	3.42 (2.99, 3.85)	2050.98 (4.81)
Cervical cancer	13 (1.29)	49	1661 (0.4)	10.7 (4.88, 16.52)	4.00 (2.89, 5.11)	563.29 (1.32)
V. Mental and behavioural disorders	80 (7.96)	47	25 932 (6.08)	65.84 (51.42, 80.27)	21.89 (19.45, 24.34)	3676.38 (8.62)
Drug-related	53 (5.27)	40	239 (0.06)	43.62 (31.88, 55.37)	103.72 (89.47, 117.97)	2736.95 (6.42)
Alcohol-related	24 (2.39)	57	589 (0.14)	19.75 (11.85, 27.66)	23.43 (18.65, 28.21)	858.2 (2.01)
IX. Diseases of the circulatory system^a^	103 (10.25)	55	145 749 (34.19)	84.78 (68.4, 101.15)	4.29 (3.87, 4.72)	3709.11 (8.7)
X. Diseases of the respiratory system	59 (5.87)	60	43 175 (10.13)	48.56 (36.17, 60.95)	4.87 (4.23, 5.5)	1989.31 (4.66)
Chronic respiratory disease^a^^b^	42 (4.18)	60	19 667 (4.61)	34.57 (24.11, 45.02)	4.63 (3.92, 5.35)	1385.96 (3.25)
XI. Diseases of the digestive system	79 (7.86)	55	14 056 (3.30)	65.02 (50.68, 79.36)	13.32 (11.83, 14.82)	2917.57 (6.84)
Alcohol-related	44 (4.38)	54.5	884 (0.21)	36.21 (25.51, 46.91)	23.4, (19.93, 27.01)	1691.42 (3.97)
Diabetes mellitus^a^	9 (0.9)	55	6989 (1.64)	7.41 (2.57, 12.25)	3.9 (2.6, 5.2)	327.39 (0.77)
XVIII. Symptoms, signs and abnormal clinical and laboratory findings, not elsewhere classified	22 (2.19)	53	16 945 (3.98)	18.11 (10.54, 25.67)	7.25 (5.7, 8.8)	892.64 (2.09)
III–IV/VI–VIII. Other non-external causes and unknown causes	67 (6.67)	53	49 161 (11.53)	55.14 (41.94, 68.35)	2.86 (2.51, 3.21)	2679.3 (6.28)
XX. External causes of morbidity and mortality	396 (39.4)	42	19 606 (4.60)	325.94 (293.83, 358.04)	17.03 (16.18, 17.89)	19 878.65 (46.61)
Accidents and poisonings with unknown intent	290 (28.86)	43	15 730 (3.69)	238.69 (211.22, 266.16)	26.72 (25.15, 28.28)	14 399.12 (33.76)
Drug poisoning	229 (22.79)	41	1162 (0.27)	188.48 (164.07, 212.9)	53.37 (49.84, 56.89)	11 690.12 (27.41)
Alcohol poisoning	15 (1.49)	53	170 (0.04)	12.35 (6.1, 18.59)	27.8 (20.62, 34.98)	609.53 (1.43)
Homicide	7 (0.7)	39	289 (0.07)	5.76 (1.49, 10.03)	7.67 (4.77, 10.56)	372.99 (0.87)
Suicide	95 (9.45)	40	3114 (0.73)	78.19 (62.47, 93.91)	8.58 (7.7, 9.46)	4932.11 (11.57)
Intentional drug poisoning	35 (3.48)	41	1062 (0.25)	28.81 (19.26, 38.35)	9.09 (7.55, 10.62)	1738.06 (4.08)
**Total (all-cause mortality)**	**1005 (100)**	**50**	**426 267 (100)**	**827.19 (776.05, 878.33)**	**5.17 (5.01, 5.34)**	**42 646.48 (100)**

CMR, crude mortality rate; SMR, standardized mortality ratio; YLL, years of life lost.

a Global leading cause of non-communicable disease (as defined by the World Health Organization).

b Smoking-related.

c Three cases of non-malign neoplasms (D00–48) were omitted from this table.

Cancers were the most common internal cause of death (*n* = 169; 17%), followed by diseases of the circulatory (*n* = 103; 10%) and digestive systems (*n* = 79; 8%). Over half of deaths from diseases in the digestive system were from an alcohol-related cause (Table 4).

Compared with age-matched controls from the general population, women with a history of incarceration were five times more likely to die from any cause (SMR: 5.17, 95% CI: 5.01–5.34) and nine times more likely to die prematurely from a preventable cause of death (SMR: 8.98, 95% CI: 8.65–9.32). The SMR was elevated across all causes of death reported in this study, including for major NCDs (SMR: 2.44, 95% CI: 2.30–2.58), which was also the leading cause of death in the general population (Table 3). The greatest difference in mortality was for any drug-induced death (SMR: 36.39, 95% CI: 34.36–38.42) (Table 3), particularly from drug-related deaths from mental or behavioural disorders (SMR: 103.72, 95% CI: 89.47–117.97) and drug poisonings with unknown intent (SMR: 53.37, 95% CI: 49.84–56.89), death from HIV or hepatitis infection (SMR: 51.6, 95% CI: 40.07–63.14; Table 4 and Supplementary Table S2, available as Supplementary data at *IJE* online) and any alcohol-related death (SMR: 24.48, 95% CI: 21.89–27.08; Table 3).

Even though <0.05% of the Norwegian adult female population ever spends time in prison, we found that 13% of all deaths from HIV or hepatitis, 11% of all drug-induced deaths and 5% of all alcohol-related deaths that occurred in any women in Norway between 2000 and 2019 were among women who had also been to prison during the same period.

Deaths among women with a history of incarceration in Norway between 2000 and 2019 accounted for a total of 42 647 YLL, of which 82.5% were lost to premature deaths that could have been avoided. A substantial number of life years were lost to drug-induced deaths (38%), followed by major NCDs (26%) and alcohol-related deaths (8%). Although drug-induced deaths and deaths from major NCDs were equally frequent, the relatively large difference in YLL is explained by the difference in median age at death (41 vs 58 years, respectively) (Table 3). For causes that were neither drug-induced, alcohol-related nor from a major NCD, suicides from other methods than intentional drug poisonings (*n* = 60, 6%) incurred the highest cost in YLL (7.5%) (Table 4).

**Figure 1.**
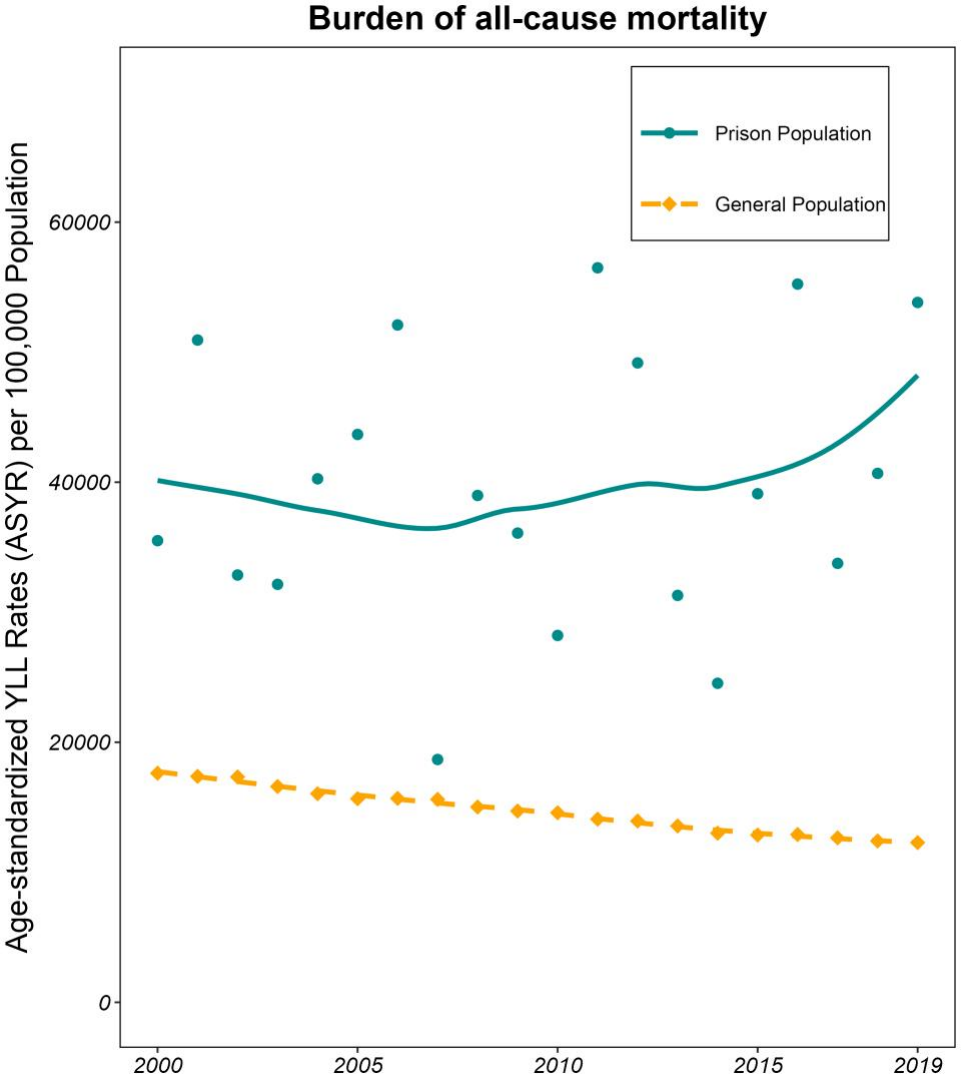
Trends of annual age-standardized years of lost life rates (ASYRs; weighted according to the age distribution of women in the Norwegian general population) based on the number of deceased from all-cause mortality (*n*_prison_ = 1005; *n*_general_ = 426 267) with a best-fitted locally estimated scatterplot smoothing (LOESS) line to assess for trends

The burden of all-cause mortality has gone down in the general population, whereas no similar trend could be seen in the annual ASYRs among women with a history of incarceration (Figure 1).

**Figure 2.**
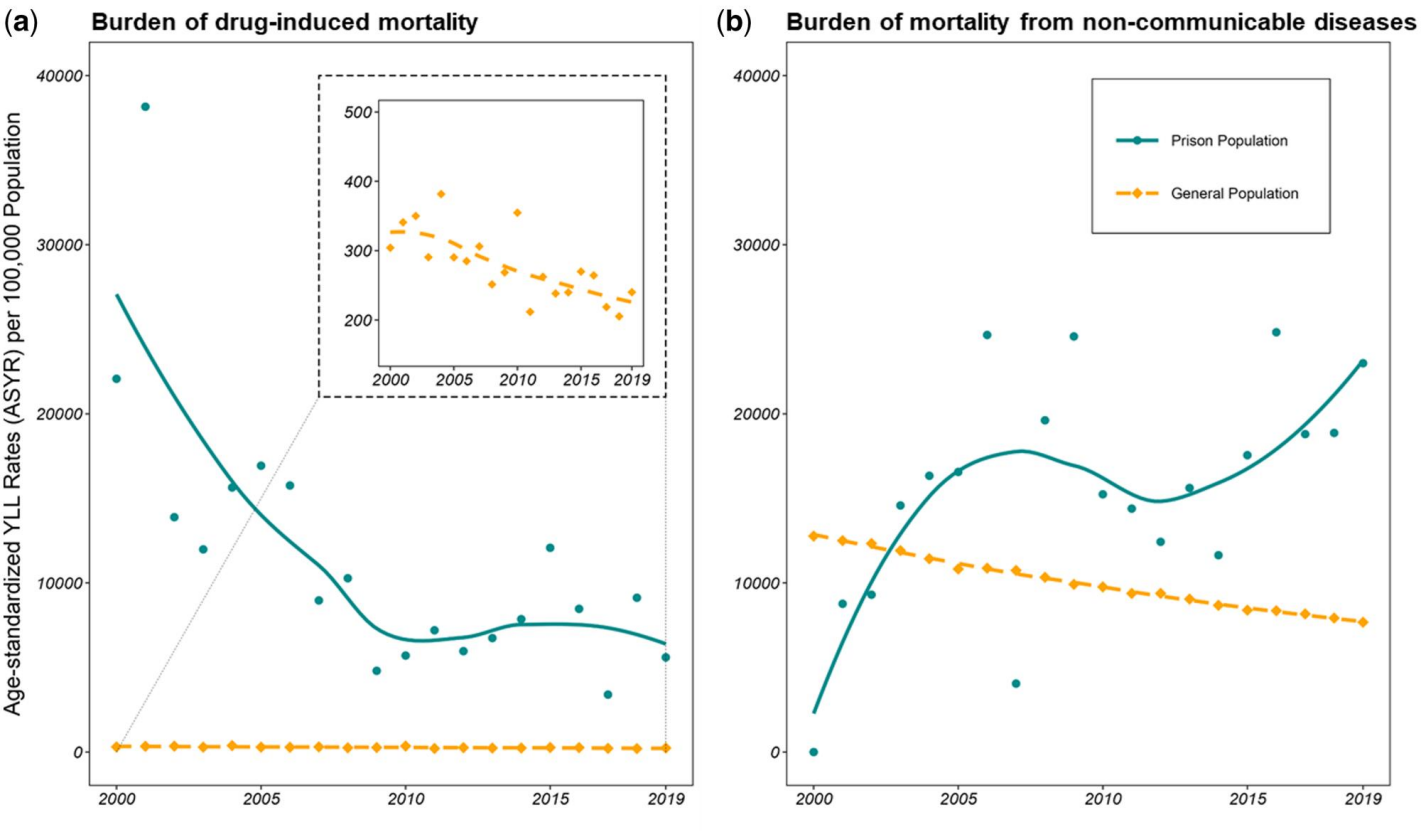
Trends of annual age-standardized years of lost life rates (ASYRs; weighted according to the age distribution of women in the Norwegian general population) plotted separately based on the number of deaths from (a) drug-induced causes (*n*_prison_ = 321; *n*_general_ =2527) and (b) alcohol use and major non-communicable diseases (*n*_prison_ = 409; *n*_general_ = 273 941) with a best-fitted locally estimated scatterplot smoothing (LOESS) line to assess for trends

Relative to the general population, the prison population had a markedly higher prevalence (73 vs 26%), risk (SMR: 36.39; Table 3) and overall burden (Figure 2a) of drug-induced deaths. However, in terms of trends over time, there are clear similarities in how the annual ASYRs from drug-induced deaths in both populations have decreased since the start of the millennium (Figure 2a).

The ASYRs for deaths from alcohol use and major NCDs, which accounted for >60% of deaths in the general population (Table 3), are more comparable (Figure 2b). However, here we observe a divergence in trends. Unlike the burden associated with drug-induced mortality, the burden associated with deaths from alcohol use and major NCDs appears to be trending upwards in the prison population. This is the reverse of the trend observed in the general population, in which the burden of mortality from these causes has steadily decreased.

## Discussion

Women with a history of incarceration are subject to a substantial burden and risk of death. Compared with their peers in the general population, they die younger, more frequently and largely from avoidable causes associated with substance use, alcohol, smoking or other NCDs. The overwhelming majority of deaths among women moving through the Norwegian prison system, occur outside of prison—following release from relatively short prison sentences. This accentuates the necessity of accessible and functional re-entry services^53^ and the role of correctional institutions as an arena for risk detection, healthcare and prevention.

High mortality among people with a history of incarceration is a worldwide phenomenon and a global public health concern.^54^^,^^55^ Compared with previous studies, the all-cause mortality rate in the Norwegian female prison population was slightly higher^26^^,^^28^^,^^30^^,^^33^^,^^56^ or similar^8^^,^^27^—with major differences most likely reflective of global variations in selection into prison and overall prison population sizes (see Supplementary Information, available as Supplementary data at *IJE* online).

To the best of our knowledge, this is the first study to apply the YLL methodology^49^ to the European prison context. Importantly, we found no evidence to suggest that the burden of general mortality in this population has gone down in the period 2000–19. This is in stark contrast to what is generally observed at the population level, both nationally and internationally.^57^ Another important finding is that the profile of causes contributing to the burden of death has changed over time. In general, we find that deaths from major NCDs (including alcohol-related deaths) has gradually replaced drug-induced causes as the primary source of lost life years; unlike in the general population, the burden of deaths from NCDs has increased, indicating that major NCDs and their lethality operate differently in these two groups.

The decline in the burden of premature mortality incurred by drug-induced causes is in line with previous research, which has documented a gradual reduction in mortality rates in the Norwegian population of persons using opioids^58^ and after release from prison.^35^ This development is likely attributable to the propagation and increased availability of opioid agonist treatment^59^ and, in the prison context, targeted efforts to reduce immediate overdose risk following release.^60^

It cannot be concluded from our data whether the trends observed in this study primarily reflect how drug use has become more survivable and thus outcompeted by other causes of death or whether there are other social or demographic factors at play. The persistently high rates of substance use disorders (SUDs), dual disorders and other mental health problems among women in Norwegian prisons^9^ nevertheless denote a population that, even when surviving one potential cause of premature death (i.e. overdose), is likely to remain at significant risk for premature mortality.^61^

### Strengths and limitations

The main strength of this study is the integrity of the underlying data material, which provided two decades of complete and reliable observations of an otherwise under-researched population. We have also applied novel and highly relevant methods of categorization (e.g. a standardized definition of premature avoidable deaths) and analysis (i.e. ASYR). Nevertheless, our results are subject to a few contextual and methodological factors that might limit their interpretability and generalizability.

First, with regard to temporal trends, the large variance in annual ASYRs might imply that the sample size (e.g. the number of deaths in the prison population) was potentially too small to sufficiently capture and determine temporal trends in mortality.

Relatedly, given the prospective nature of our study, we were also more likely to record a drug-induced death (which is often an acute event with an increased likelihood of occurring shortly after release from prison) than a death from an NCD (which, in addition to acute events such as strokes, typically involves more slowly operating causes such as cancer or other chronic disorders) in the first years of the observation period.

Moreover, the rates of overdose deaths in Norway were particularly high in the years 2000–03,^62^ which also marks the beginning of our observation period. Not knowing whether the peak observed in our own data was at the tail of a general historic downwards trend in opioid-related deaths among women with a history of incarceration or whether it represented an anomaly in an otherwise flatter curve in drug-induced mortality encourages some caution in how this apparently dramatic reduction should be understood.

In sum, any temporal change presented or discussed in this paper should be considered exploratory and with these limitations in mind.

Second, some causes of death are potentially misclassified or insufficiently classified^63^ in death registry data. For instance, deaths involving injury or poisonings with unknown intent might be incorrectly classified as accidents instead of suicides and vice versa. Although the NDR holds high-quality data^44^ and has been assessed as having good reliability and validity for suicide classification,^64^ the potential for misclassification does introduce some level of uncertainty, particularly surrounding the prevalence of suicide deaths.

Moreover, in the case of differential misclassification between study cohorts, comparative analyses might be subject to substantial bias.^65^ Given the demographic and risk-profile discrepancies between deceased women with a history of incarceration and those in the general population (e.g. age and risk of violent death, which in turn might influence the degree of post-mortem examination and precision of cause classification), it is possible that some of our results (e.g. SMRs) are biased in the direction of detecting exaggerated group differences in certain cause-specific mortalities.

Third, results might not generalize to contexts or countries outside of Scandinavia or northern Europe (see Supplementary Information, available as Supplementary data at *IJE* online).

### Implications

Our results provide an extensive description of mortality among women with a history of incarceration in Norway and how this highly marginalized population still dies both excessively and prematurely, albeit slightly later and from other (avoidable) causes compared with two decades ago. Although women with a history of incarceration represent a very small minority, we found that deaths in this population represented a sizeable number of avoidable deaths at the national level. We believe this highlights the potential—and responsibility—of prison institutions to act as stakeholders in the management and promotion of public health.

For at-risk women living on the margins of society, interactions with the correctional system might represent one of the few points of extended contact with public services and the healthcare system.^66^ Although it is debatable to what extent prison institutions provide the optimal environment for supporting women presenting with severe mental health and substance use problems,^20^^,^^67^^,^^68^ imprisonment and subsequent re-entry programmes nevertheless represents a unique window of opportunity to detect and address health issues and thus to reduce morbidity and mortality in highly marginalized persons and communities.^69^

It is our hope that the risks and vulnerabilities identified in this study will both contribute to increased awareness and inform policy change to reduce the risk of premature deaths among women who have been incarcerated. As demonstrated by the apparent successes of the national overdose prevention strategy^60^ in drawing attention to drug-induced mortality following release from prison, such initiatives are both viable and potentially effective. However, continued efforts to prevent mortality from acute events such as drug poisonings and suicides are still essential in this highly vulnerable population. In addition, the equally excessive burden of premature deaths from somatic and other lifestyle-related causes calls for a broader spectrum of targeted health and social interventions in order to meaningfully reduce mortality at a time in which drug-induced deaths are already more effectively prevented or delayed.

## Ethics approval

The study was approved by the Regional Committees for Medical Research Ethics South East Norway (2012/140) and was approved as being exempt from informed consent. Data files were linked by the Norwegian Institute of Public Health, which also created and stored the linkage code. All the methods were carried out in accordance with relevant guidelines and regulations (such as Declaration of Helsinki).

## Supplementary Material

dyae032_Supplementary_Data

## Data Availability

This population study used individual-level data from the Norwegian Prison Registry (held by the Directorate of Norwegian Correctional Service) and the Norwegian Cause of Death Registry (held by the Norwegian Institute of Public Health). The ethical approval of this research project does not include permission to share the raw data publicly. Qualifying researchers can apply for access to relevant data from the Norwegian Institute of Public Health and the Directorate of Norwegian Correctional Service on approval from the Regional Committees for Medical and Health Research Ethics.
