# Correction to: Mortality in women with a history of incarceration in Norway: a 20-year national cohort study

**DOI:** 10.1093/ije/dyae155

**Published:** 2024-11-12

This is a correction to: Vegard G Svendsen, Anne Bukten, Torbjørn Skardhamar, Marianne Riksheim Stavseth, Mortality in women with a history of incarceration in Norway: a 20-year national cohort study, *International Journal of Epidemiology*, Volume 53, Issue 2, April 2024, https://doi.org/10.1093/ije/dyae032

In the originally published version of the manuscript, a detail of funding was omitted. Part of the first sentence of the **Funding** section is now corrected to read: “[…] and the Norwegian Research Council (301535 and 300995).” instead of: “[…] and the Norwegian Research Council (301535).”

