# Supplementary material for: Mortality in women with a history of incarceration in Norway: a 20-year national cohort study
Source: Int J Epidemiol. 2024 Mar 13;53(2):dyae032. doi: 10.1093/ije/dyae032 (PMC10937902; doi:10.1093/ije/dyae032)
Supplement: dyae032_Supplementary_Data [file dyae032_supplementary_data.docx]

**Supplementary material**

Contents

[Supplementary Information 1](#_Toc156995498)

[Setting - Elaboration on the Norwegian prison context 1](#_Toc156995499)

[Definitions and sources of cause of death categories 1](#_Toc156995500)

[Extended discussion of comparison of results with other studies/countries 2](#_Toc156995501)

[Figures and Tables 3](#_Toc156995502)

[Figure S1: Trends of age-standardized years of lost life rate 3](#_Toc156995503)

[Table S1: Demographic characteristics by leading causes of death 3](#_Toc156995504)

[Table S2: Mortality statistic for hepatitis and HIV 3](#_Toc156995505)

[References 3](#_Toc156995506)

# Supplementary Information

## Setting - Elaboration on the Norwegian prison context

Norway has a low imprisonment rate (58/100 000), which falls well below the European average (124/100 000) (1) and the US (531/100 000)(2). Women account for about 6.5% of the Norwegian total prison population, which is high in the European context but lower than in the US. The country’s correctional philosophy is motivated by rehabilitation and successful reintegration, and the incarcerated population has the same rights to universal health care as the general population (3).

## Definitions and sources of cause of death categories (avoidable premature mortality; drug-induced and alcohol-related deaths; and smoking related deaths)

Avoidable premature mortality was defined according to the Organisation for Economic Co-Operation and Development (OECD) and the European Commission, Directorate for Social statistics (EUROSTAT) definitions for preventable deaths and deaths from treatable causes in persons under the age of 75 (4). Causes of death that were classified by OECD and EUROSTAT as an equal combination of both treatable and preventable, were categorized as preventable deaths. We identified drug-induced and alcohol-related deaths based on the classifications developed by the Australian National Drug and Alcohol Research Centre (NDARC) (5). Mortality from major NCDs (cardiovascular disease, cancer, diabetes mellitus and chronic respiratory disease) were reported according to the global health indicators of the World Health Organization (WHO) and the United Nations Economic and Social Council (ECOSOC) (6).

To the best of our knowledge, there exists no established framework for defining smoking-related mortality based on death registries alone. Because we did not have access to smoking prevalence data and the extensive list of diseases associated with smoking (7) would be overly inclusive in defining smoking-related deaths, mortality related to smoking was limited to those major NCDs most strongly associated with mortality in currently and formerly smoking females relative to non-smokers (i.e., lung cancer, laryngeal cancer and chronic respiratory disease) (8, 9).

## Extended discussion of comparison of results with other studies/countries

We note several points of both overlap and departure from other immediately relevant studies. Our findings are for instance highly congruent with those reported by Graham et al., from a similarly comprehensive mortality study from Scotland (10). On the other hand, crude all-cause mortality rates were slightly lower in our study compared to a national cohort study from Sweden, which also had much higher rates of suicide deaths (11). Suicide occurred at a lower rate and had a lower SMR in our study compared to pooled estimates from a recent systematic review and meta-analysis (12) but was still highly prevalent and accounted for almost 10% of all deaths and more than one tenth of lost life years.

All cause-mortality was slightly higher in our population compared to what has been reported from Canada (13), United States (14), Brazil (15) and Australia (16, 17).

The higher mortality in European prison populations relative to countries such as the US are likely, at least in part, a reflection of important differences in selection into prison and overall prison population size. It should also be noted that, in addition to these contextual and demographic differences that exist between study populations in this literature, there are also important methodological differences in how mortality is measured across studies (18).

# Figures and Tables

## Figure S1: Trends of age-standardized years of lost life rate, plotted separately based on the number of deaths from (a) alcohol use drug-induced causes (n_prison_ = 410; n_general_ = 4245) and (b) deaths from alcohol use and major non-communicable diseases (n_prison_ = 320; n_general_ = 272 223) with a best fitted locally estimated scatterplot smoothing (LOESS) line to assess for trends


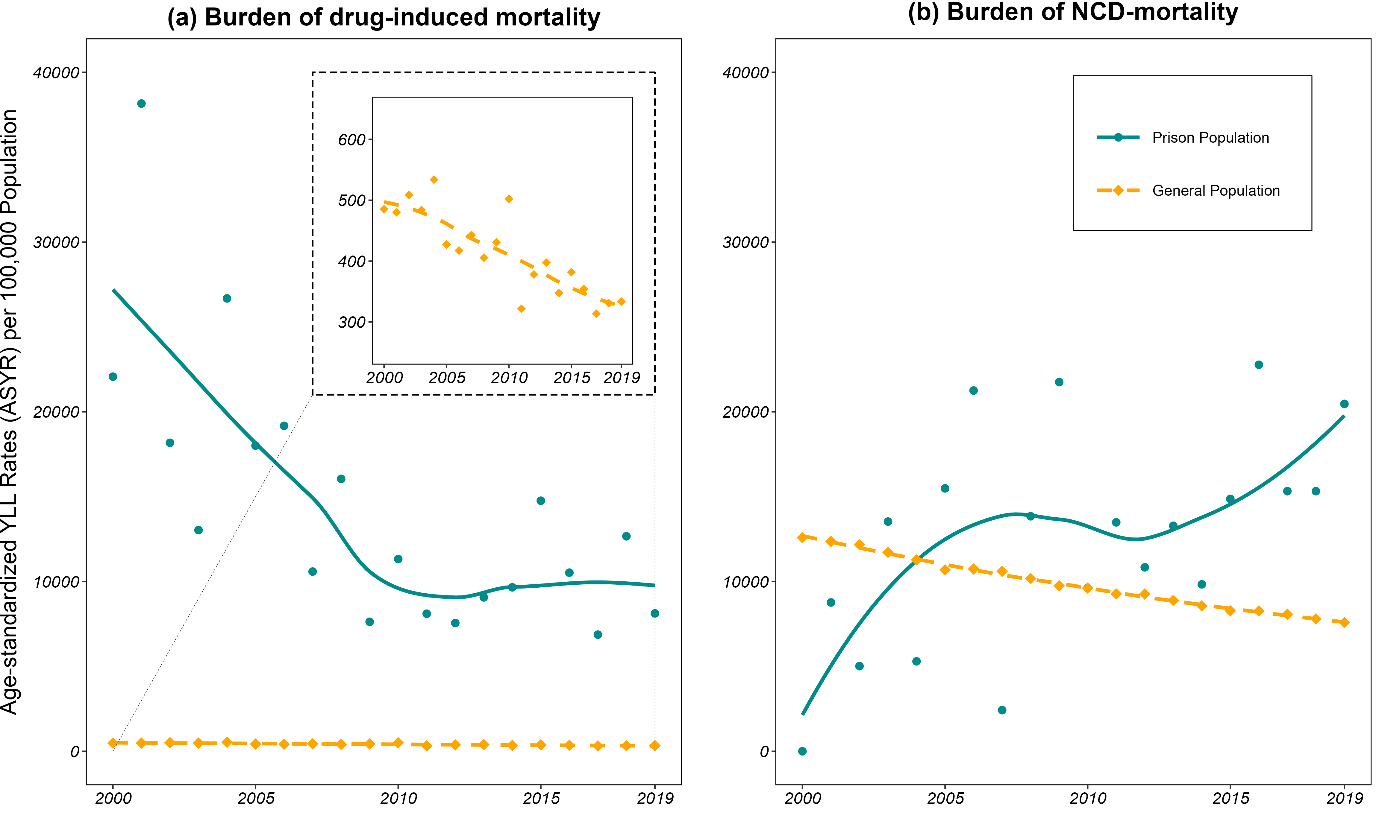


NCD, non-communicable disease.

## Table S1: Demographic characteristics by leading causes of death

|  | **Age at first recorded incarceration^a^** | **Sentence length (days)^a^** | **History of more than one incarceration^b^** |
| --- | --- | --- | --- |
| **Avoidable premature mortality** |  |  |  |
| Deaths from preventable causes | 39 (31, 48) | 28 (19, 56) | 205 (28) |
| Deaths from treatable causes | 44 (37, 50) | 27 (20, 52) | 25 (30) |
| **Mortality from substance use and NCDs** |  |  |  |
| Drug induced deaths (excluding alcohol) | 33 (28, 39) | 32 (20, 62) | 105 (33) |
| Alcohol related deaths | 46 (40, 51) | 25 (18, 36) | 20 (22) |
| Major NCDs | 48 (41, 55) | 25 (18, 38) | 65 (20) |
| ^a^Median (Interquartile range); ^b^n (%) NCDs, non-communicable diseases. | | | |

## Table S2: Mortality statistic for hepatitis and HIV

| **Cause of death** | **No. of Deaths** | **CMR (95% CI)** | **SMR (95% CI)** |
| --- | --- | --- | --- |
| Hepatitis | 11 | 9.05 (3.7, 14.4) | 62.73 (43.82, 81.65) |
| *B* | *1* |  |  |
| *Unknown* | *10* |  |  |
| HIV | 9 | 7.41 (2.57, 12.25) | 42.41 (28.27, 56.55) |
| CMR, crude mortality rate. SMR, standardized mortality ratio. | | | |

# References

1. Aebi MF, Tiago MM. SPACE I - 2020 – Council of Europe Annual Penal Statistics: Prison populations. Strasbourg: Council of Europe; 2021.

2. World Prison Brief. World Prison Brief (WPB) - United States of America n.d [Available from: https://www.prisonstudies.org/country/united-states-america.

3. Pratt J. Scandinavian Exceptionalism in an Era of Penal Excess: Part I: The Nature and Roots of Scandinavian Exceptionalism. The British Journal of Criminology. 2008;48(2):119-37.

4. OECD/Eurostat. Avoidable mortality: OECD/Eurostat lists of preventable and treatable causes of death (January 2022 version). 2022.

5. Santo T, Bharat C, Colledge-Frisby S, et al. Mortality among people with substance use disorders: A toolkit for classifying major causes of death. National Drug and Alcohol Research Centre: UNSW Sydney; 2022.

6. World Health Organization. Noncommunicable Diseases: Fact sheets on sustainable development goals - health targets. Denmark: WHO Regional Office for Europe; 2017.

7. Lushniak BD, Samet JM, Pechacek TF, Norman LA, Taylor PA. The Health consequences of smoking—50 years of progress : a report of the Surgeon General. United States: Public Health Service. Office of the Surgeon General. National Center for Chronic Disease Prevention and Health Promotion. Office on Smoking and Health; 2014.

8. Lariscy JT, Hummer RA, Rogers RG. Cigarette Smoking and All-Cause and Cause-Specific Adult Mortality in the United States. Demography. 2018;55(5):1855-85.

9. Binswanger IA, Carson EA, Krueger PM, Mueller SR, Steiner JF, Sabol WJ. Prison tobacco control policies and deaths from smoking in United States prisons: population based retrospective analysis. BMJ. 2014;349:g4542.

10. Graham L, Fischbacher CM, Stockton D, Fraser A, Fleming M, Greig K. Understanding extreme mortality among prisoners: a national cohort study in Scotland using data linkage. Eur J Public Health. 2015;25(5):879-85.

11. Chang Z, Lichtenstein P, Larsson H, Fazel S. Substance use disorders, psychiatric disorders, and mortality after release from prison: a nationwide longitudinal cohort study. The Lancet Psychiatry. 2015;2(5):422-30.

12. Janca E, Keen C, Willoughby M, et al. Sex differences in suicide, suicidal ideation, and self-harm after release from incarceration: a systematic review and meta-analysis. Social Psychiatry and Psychiatric Epidemiology. 2022.

13. Kouyoumdjian FG, Kiefer L, Wobeser W, Gonzalez A, Hwang SW. Mortality over 12 years of follow-up in people admitted to provincial custody in Ontario: a retrospective cohort study. CMAJ Open. 2016;4(2):E153-61.

14. Binswanger IA, Blatchford PJ, Mueller SR, Stern MF. Mortality After Prison Release: Opioid Overdose and Other Causes of Death, Risk Factors, and Time Trends From 1999 to 2009. Annals of Internal Medicine. 2013;159(9):592-600.

15. Liu YE, Lemos EF, Goncalves CCM, et al. All-cause and cause-specific mortality during and following incarceration in Brazil: A retrospective cohort study. PLoS Med. 2021;18(9):e1003789.

16. van Dooren K, Kinner SA, Forsyth S. Risk of death for young ex-prisoners in the year following release from adult prison. Aust N Z J Public Health. 2013;37(4):377-82.

17. Kariminia A, Butler T, Corben S, et al. Extreme cause-specific mortality in a cohort of adult prisoners--1988 to 2002: a data-linkage study. Int J Epidemiol. 2007;36(2):310-6.

18. Kinner SA, Forsyth S, Williams G. Systematic review of record linkage studies of mortality in ex-prisoners: why (good) methods matter. Addiction. 2013;108(1):38-49.
